# Supplementary material for: Aberrant expression of CITED2 promotes prostate cancer metastasis by activating the nucleolin-AKT pathway
Source: Nat Commun. 2018 Oct 5;9:4113. doi: 10.1038/s41467-018-06606-2 (PMC6173745; doi:10.1038/s41467-018-06606-2)
Supplement: Supplementary file 1 — Supplementary Information [file 41467_2018_6606_MOESM1_ESM.pdf]

Supplementary information

Aberrant expression of CITED2 promotes prostate cancer metastasis by activating the nucleolin-AKT pathway

Shin *et al.*

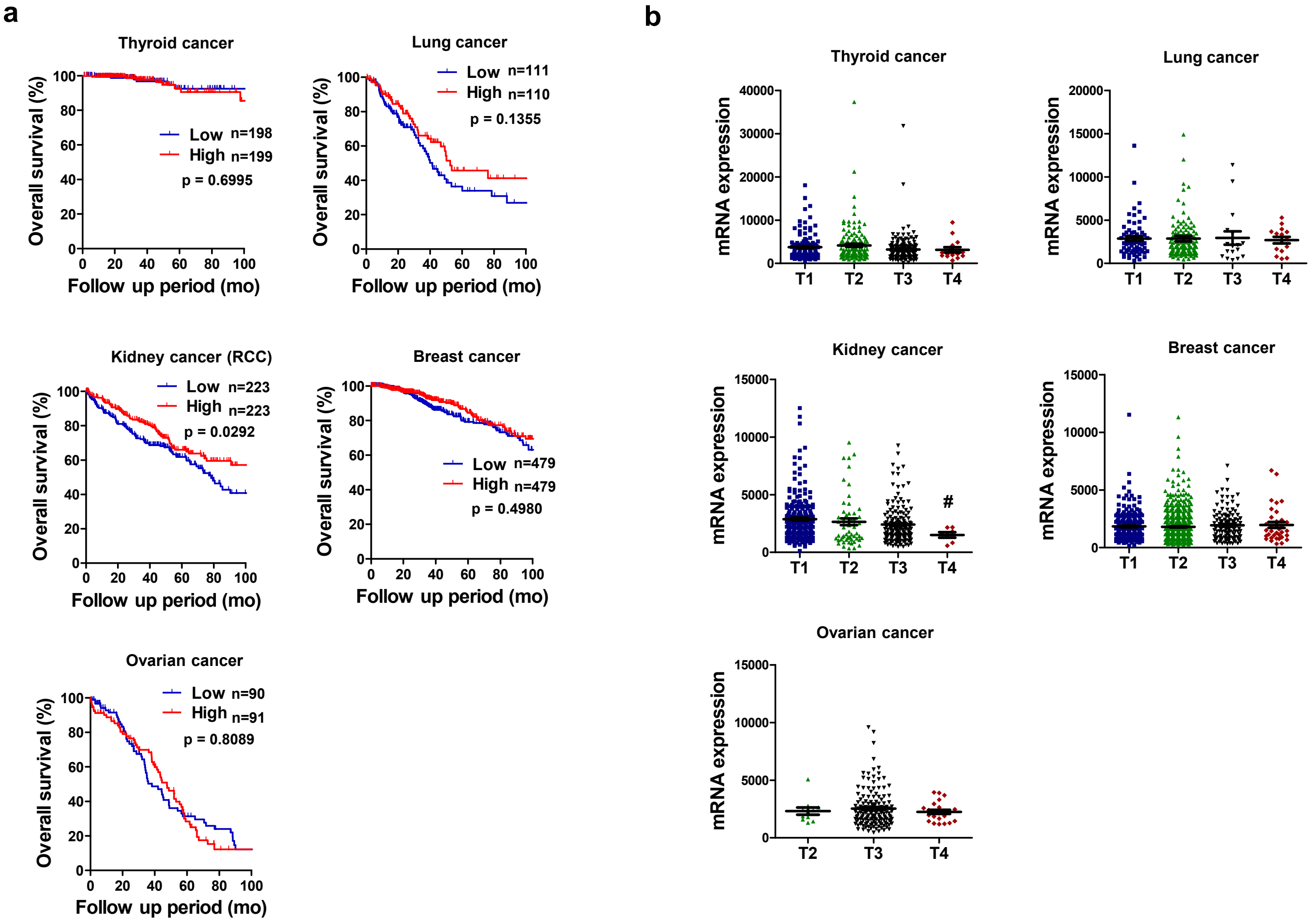

Supplementary Fig. 1 CITED2 expression does not correlate with the overall survival and the tumor stage in thyroid, ovarian, lung, and breast cancer. Data were obtained from using The Cancer Genome Atlas (TCGA). **a** Kaplan-Meier overall survival analysis of thyroid, lung, kidney, breast and ovarian cancer patients. P-value was calculated by Log-rank test. **b** CITED2 mRNA levels according to tumor stages in human thyroid, lung, kidney, breast, and ovarian cancer tissues. Dot plots indicate the distribution of values, and the horizontal lines are the mean ± s.e.m. # denotes $P < 0.05$ versus the T1 group by Mann-Whitney statistical analysis. T1, blue squares; T2, green triangles; T3, black inverted triangles; T4, red diamond.

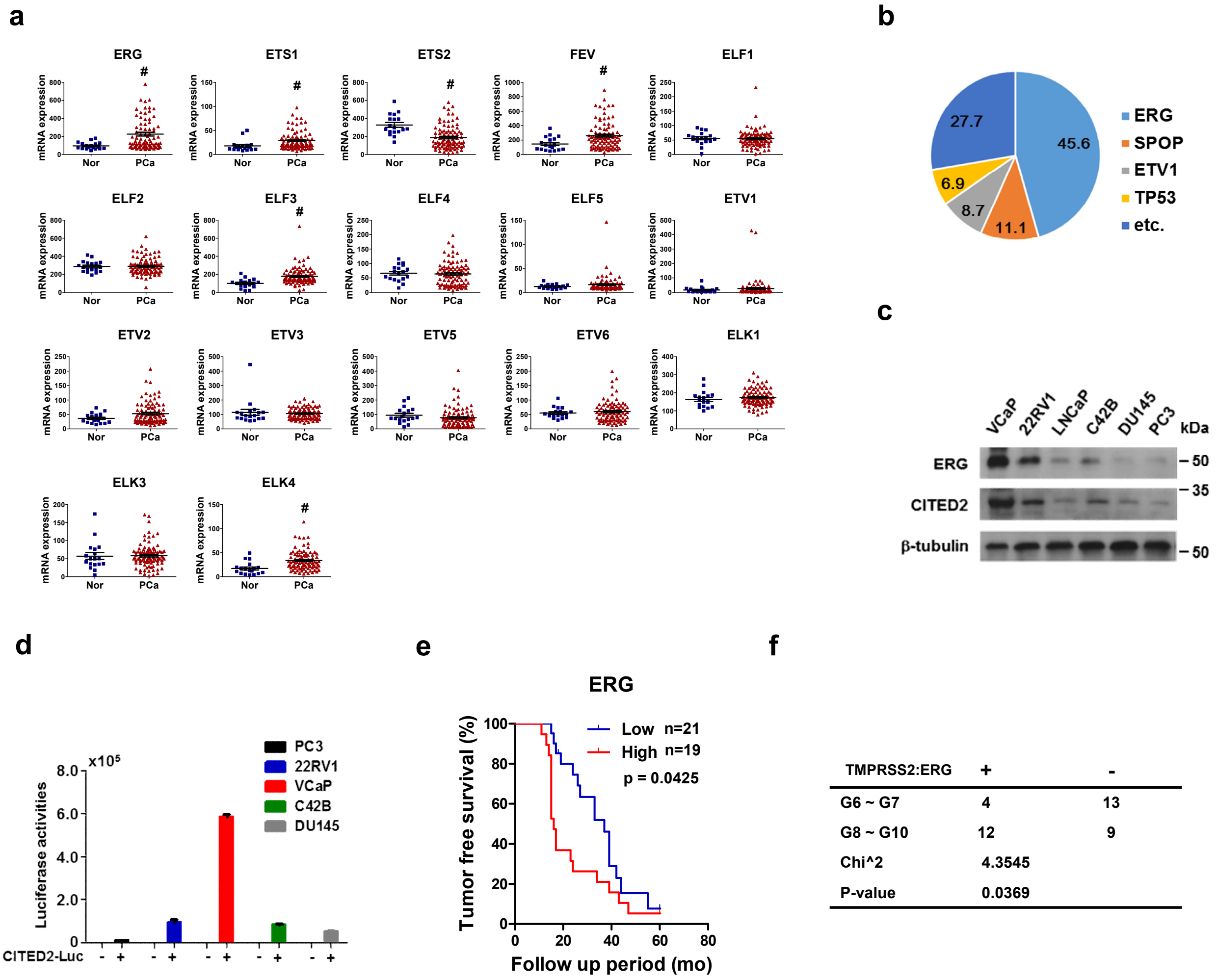

TMPRSS2:ERG	+	-
G6 ~ G7	4	13
G8 ~ G10	12	9
Chi^2	4.3545	
P-value	0.0369	

Supplementary Fig. 2 ERG is upregulated due to the TMPRSS2-ERG gene fusion in prostate cancer. **a** Comparison of ETS gene family mRNA expression between normal and cancer prostate tissues, which were obtained from data set GES6919. Nor, normal prostate tissue (N = 19, blue squares); PCa, prostate tumor tissue (N = 90, red triangles). The horizontal lines represent the mean ± s.e.m., # denotes P < 0.05 versus the normal prostate group by Mann-Whitney statistical analysis. **b** The circular chart shows mutation subtypes identified in 333 prostate cancer patients. Data were obtained from TCGA (Prostate Adenocarcinoma, Cell 2015). **c** Lysates of prostate cancer cell lines were immunoblotted with anti-ERG, anti-CITED2, or anti-β-tubulin antibody. **d** The activities of the CITED2 promoter-luciferase reporter. Prostate cancer cells were transfected with the reporter plasmid. Luciferase activities (the mean + s.d., n=3) were measured by a luminometer. **e** Kaplan-Meier tumor-free survival analysis of prostate cancer patients. P-value was calculated by Log-rank Test. **f** Distribution of the TMPRSS2:ERG fusion according to the prostate cancer Gleason score was statistically analyzed using chi-square test.

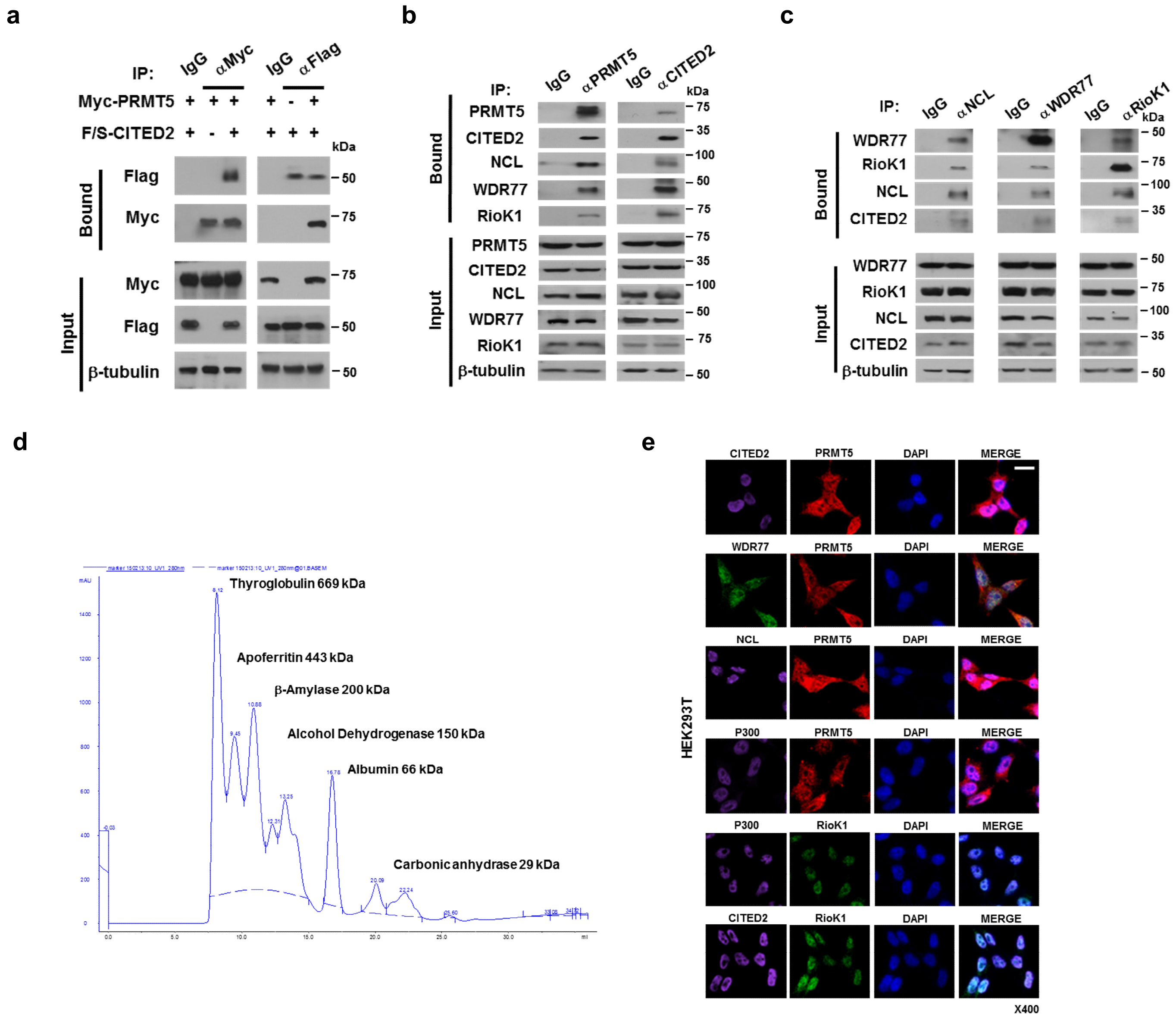

Supplementary Fig. 3 CITED2 is included in the multimeric complex of NCL. **a** HEK293T cells were cotransfected with Flag/SBP-CITED2 and Myc-PRMT5. Proteins in cell lysates were immunoprecipitated with anti-Myc, anti-Flag, or IgG, and the precipitates were immunoblotted with the indicated antibodies. **b** HEK293T cell lysates were immunoprecipitated with anti-PRMT5 antibody or anti-CITED2 or IgG, and the precipitates were immunoblotted with the indicated antibodies. **c** HEK293T cell lysates were immunoprecipitated with anti-NCL, anti-WDR77, anti-RioK1 antibody, or IgG, followed by immunoblotting with the indicated antibodies. **d** Standard protein markers used for FPLC analysis with Superdex™ 200 column. **e** Representative immunofluorescence images. HEK293T cells were grown on coverslips, fixed with methanol, and stained with the indicated antibodies. All samples were stained with DAPI to visualize nuclei. The scale bar represents 20 μm.

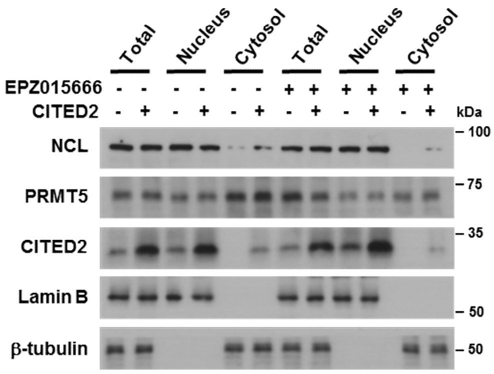

Supplementary Fig. 4 Translacation of NCL was decreased by a PRMT5 inhibitor. HEK293T cells were transfected with pcDNA or CITED2 with EPZ015666 (5 μM), and the cell lysates were fractionated to cytosoli and nuclear components. The cell fractions were immunoblotted with the indicated antibodies.

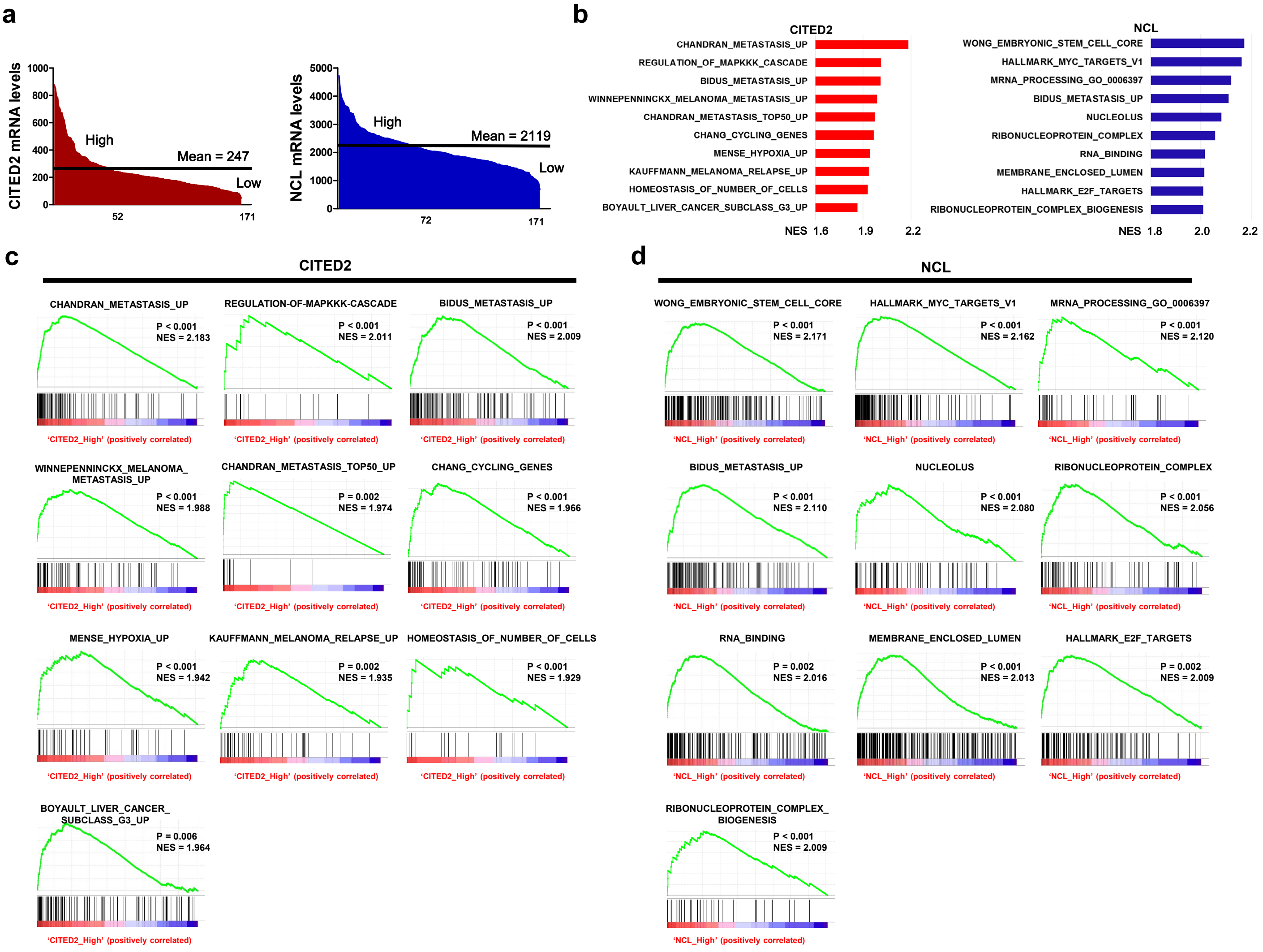

Supplementary Fig. 5 CITED2 and NCL both are positively associated with high expression of metastasis-related gene sets. **a** Distribution of CITED2 (left) or NCL (right) mRNA levels in 171 human prostate tissues (data set GSE6919). Based on the mean values, the prostate cancer tissues were divided into high and low groups. The numbers of cancers are 52 in the CITED2 high, 119 in the CITED2 low, 72 in the NCL high, and 99 in the NCL low group. **b** Top 10 gene sets showing a positive correlation with CITED2 (left) or NCL (right) are ranked. **c, d** Enrichment plots of the top 10 gene sets that were positively correlated with CITED2 or NCL expression with P < 0.05 and FDR < 0.30.

a

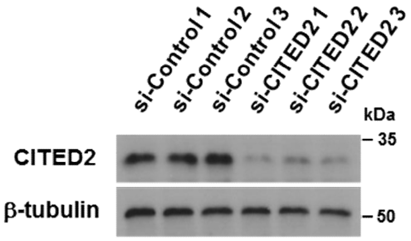

b

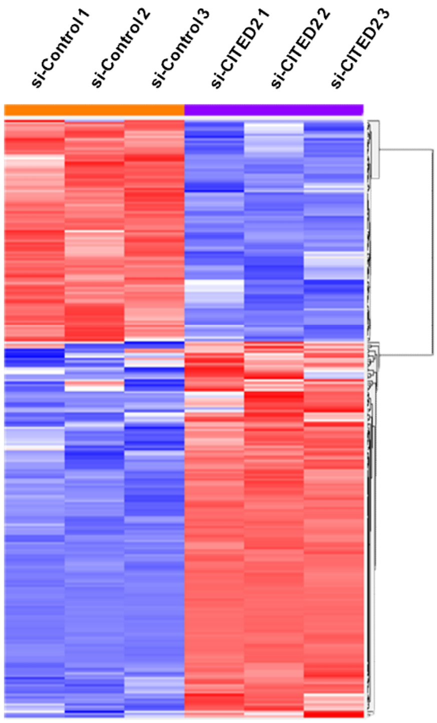

Supplementary Fig. 6 CITED2 promotes metastasis-related gene sets in PC3 cells **a** CITED2 was immunoblotted to verify knockdown. **b** Heat map of genes that were differentially expressed in si-Control group (n = 3) and si-CITED2 group (n = 3) with P-value < 0.05 and Fold change > 2.

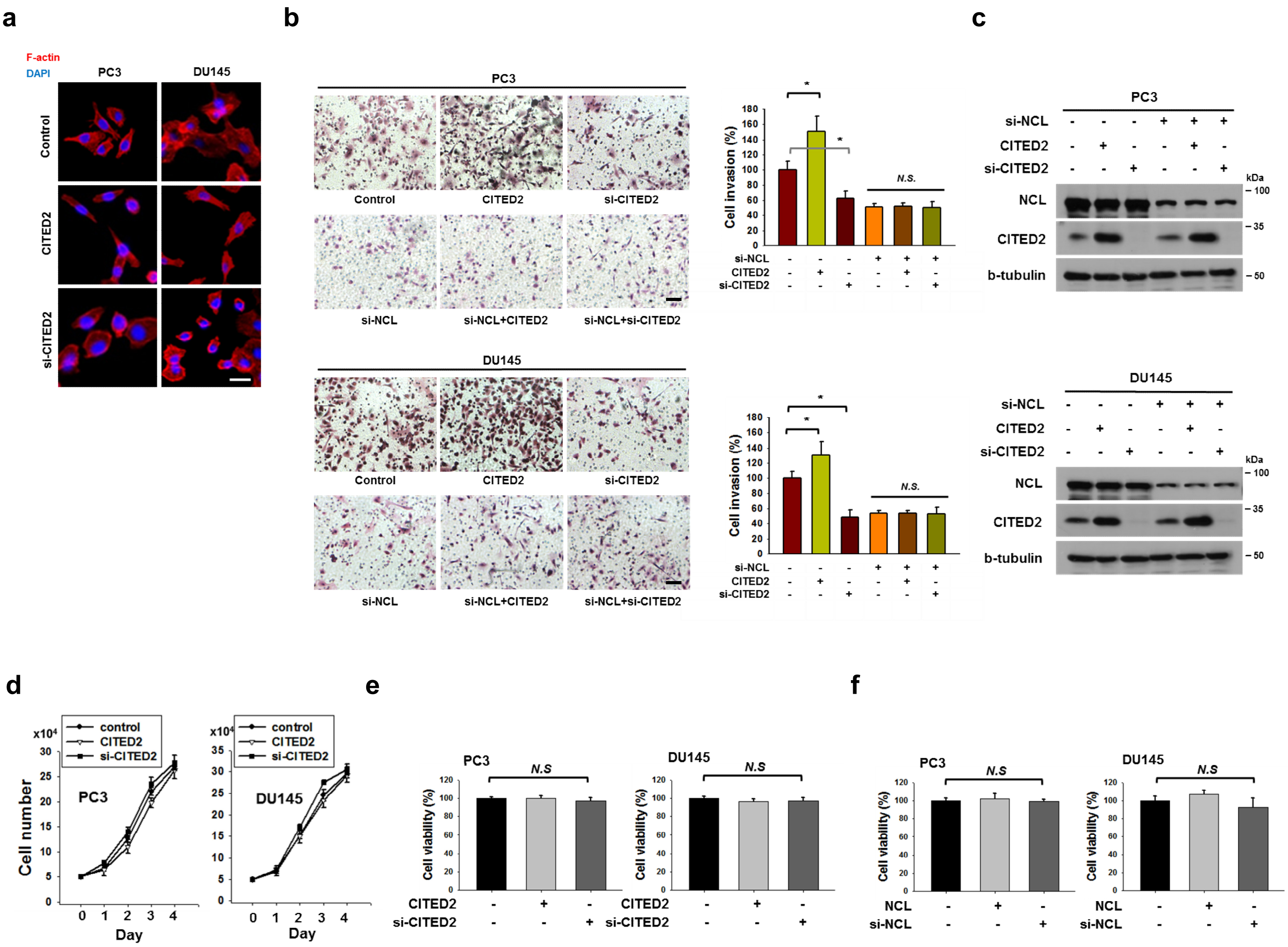

Supplementary Fig. 7 CITED2 promotes prostate cancer cell invasion NCL-dependently. **a** PC3 and DU145 cells were transfected with CITED2 or si-CITED2, and stained with rhodamine phalloidin (red) and DAPI (blue). The scale bar represents 20μm. **b** Representative trans-well membrane pictures of invasion assay. The transfected PC3 and DU145 cells ($1x10^4$ per well) were loaded in the upper chamber of the trans-well culture dish. After 12 h, cells passing through a Matrigel-coated interface membrane were stained (left) and counted (right). Cells were stained and counted, which are presented as bar graphs. The scale bar represents 25 μm. **c** CITED2 and NCL were immunoblotted to verify knockdown and overexpression. **d** Cell growth curves. PC3 or DU145 cells, which had been transfected with CITED2 or si-CITED2, were plated in 24-well culture dishes (5×10^4 per well) and counted at the indicated time. **e** Cell viabilities were measured using MTT. **f** Cell viabilities were measured using MTT. All results in the above graphs are presented as the means ± s.d. from three independent experiments and * denotes $P < 0.05$ versus the indicated control groups by Student's t-test and N.S. does 'not significantly different' among the groups.

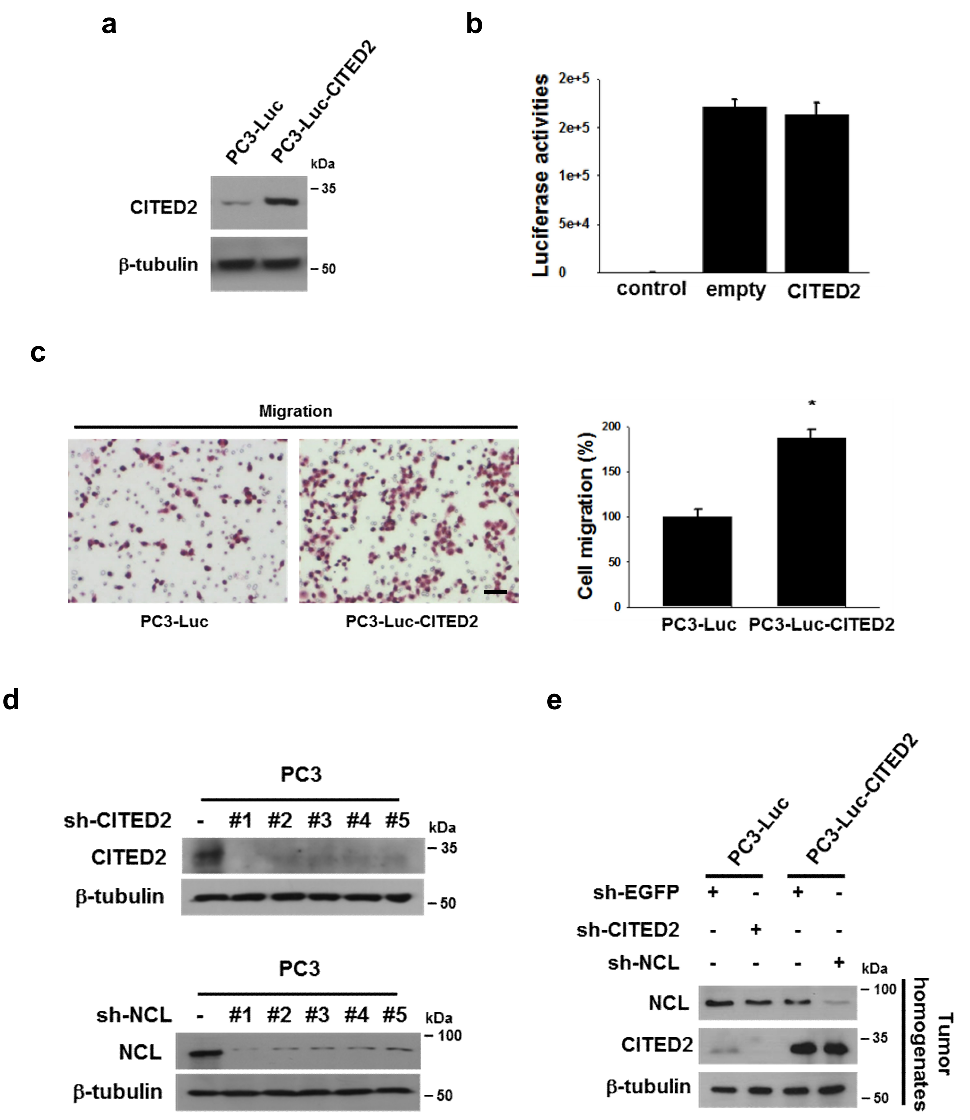

Supplementary Fig. 8 Verification of PC3 stable cell lines. **a** CITED2 proteins were immunoblotted in a PC3 stable cell line transfected with Luc-CITED2. **b** The luciferase activities in PC3 stable cell lines were measured using a luminometer. Each bar represents the mean + s.d., from 3 experiments. **c** Representative pictures of trans-well membrane migration assays in PC3-Luc and PC3-Luc-CITED2 stable cell lines (left). Migrated cells were stained and counted (right). Each bar represents the mean + s.d., from 3 experiments. * denotes P < 0.05 versus the indicated control groups by Student's t-test. The scale bar represents 25 μm. **d** Efficacies of five different sequence shRNAs (#1 - #5) targeting CITED2 or NCL in PC3 cell lysates. Cell lystaes were immunoblotted with anti-CITED2 or anti-NCL antibody. The shCITED2 #4 and the shNCL #1 were used in xenograft experiments. **e** CITED2 and NCL proteins were immunoblotted in tumor homogenates from mice.

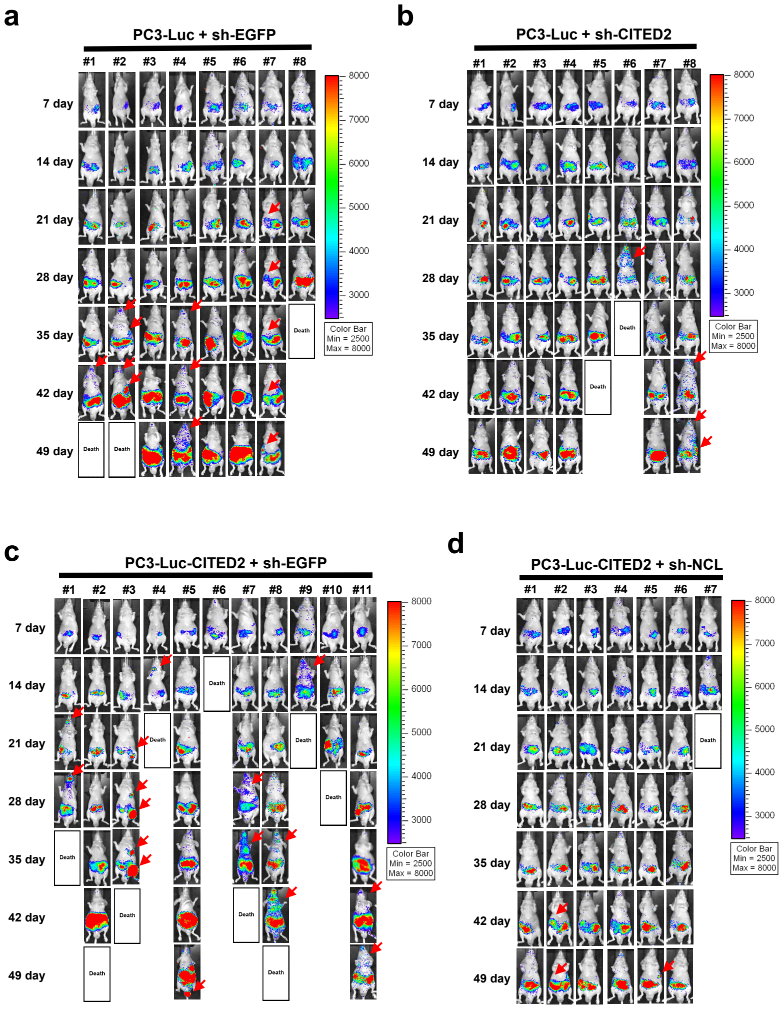

Supplementary Fig. 9 Bioluminescence images of mice having orthotopically grafted prostate cancer. Luciferase-expressing PC3 stable cell lines were injected into the prostates of mice. Bioluminescence images of live mice were monitored using Xenogen IVIS® Lumina 100 weekly for 7 weeks. Red arrows indicate metastasis tumors. Color scale bars represent uminescence intensity from purple (low) to red (high).

a

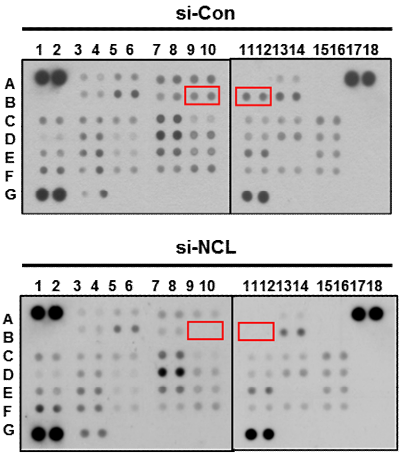

b

	1	2	3	4	5	6	7	8	9	10	11	12	13	14	15	16	17	18
A	Reference		p38a (T180/Y182)		ERK1/2 (T202/Y204, T185/Y187)		JNK1/2/3 (T183/Y185, T221/Y223)		GSK-3a/b (S21/S9)				p53 (S392)				Reference	
B			EGFR (Y1086)		MSK1/2 (S376/S360)		AMPKa1 (T183)		Akt 1/2/3 (S473)		Akt 1/2/3 (T308)		p53 (S46)					
C	TOR (S2448)		CREB (S133)		HSP27 (S78/S82)		AMPKa2 (T172)		b-Catenin		p70 S6 Kinase (T389)		p53 (S15)		c-Jun (S63)			
D	Src (Y419)		Lyn (Y397)		Lck (Y394)		STAT2 (Y689)		STAT5a (Y694)		p70 S6 Kinase (T421/S424)		RSK1/2/3 (S380/S386/S377)		eNOS (S1177)			
E	Fyn (Y420)		Yes (Y426)		Fgr (Y412)		STAT6 (Y641)		STAT5b (Y699)		STAT3 (Y705)		p27 (T198)		PLC-g1 (Y783)			
F	Hck (Y411)		Chk-2 (T68)		FAK (Y397)		PDGF Rb (Y751)		STAT5a/b (Y694/Y699)		STAT3 (Y727)		WNK1 (T60)		PYK2 (Y402)			
G	Reference		PRAS40 (T246)						PBS		HSP60						PBS	

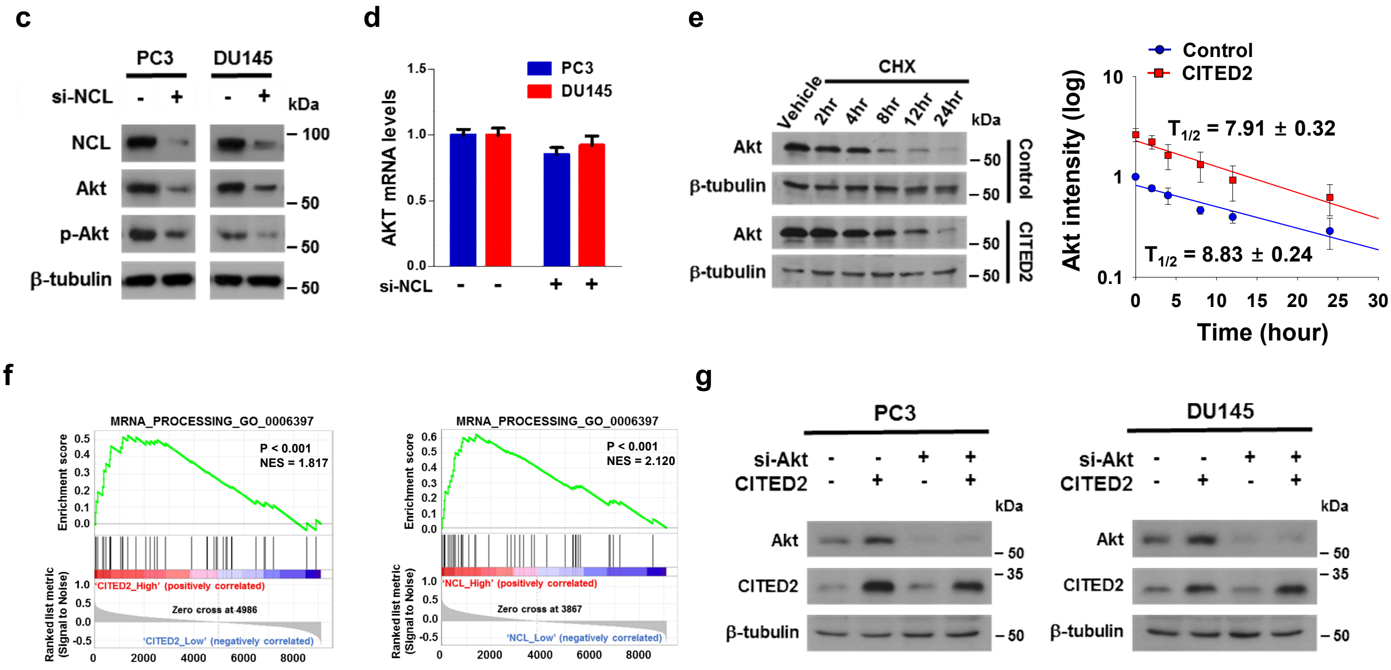

Supplementary Fig. 10. CITED2 enhances AKT synthesis by promoting mRNA translation. **a** PC3 cells were transfected with si-Con or si-NCL, and the cell lysates were subjected to the Proteome Profiler™ phospho-kinase array kit. The red boxes indicate blots of AKT phosphorylated at T308 and S473. **b** Protein names and phosphorylation sites in the Proteome Profiler™ human phosphor-kinase array kit. **c** Immunoblotting of NCL, AKT, p-AKT and b-tubulin in PC3 and DU145 cells that were transfected with si-Con or si-NCL. **d** The AKT mRNA levels in PC3 and DU145 cells were measured by RT-qPCR. **e** DU145 cells, which had been transfected with pcDNA or CITED2, were treated with 100 μM cycloheximide, and then cells were incubated for the indicated times. AKT levels were detected by Western blotting and quantified using ImageJ. **f** Representative enrichment plots of the mRNA processing gene set which commonly correlates with CITED2 (left) and NCL (right). **g** AKT and CITED2 proteins were immunoblotted to verify knockdown and overexpression. All results in the above graphs are presented as the means ± s.d. from three independent experiments.

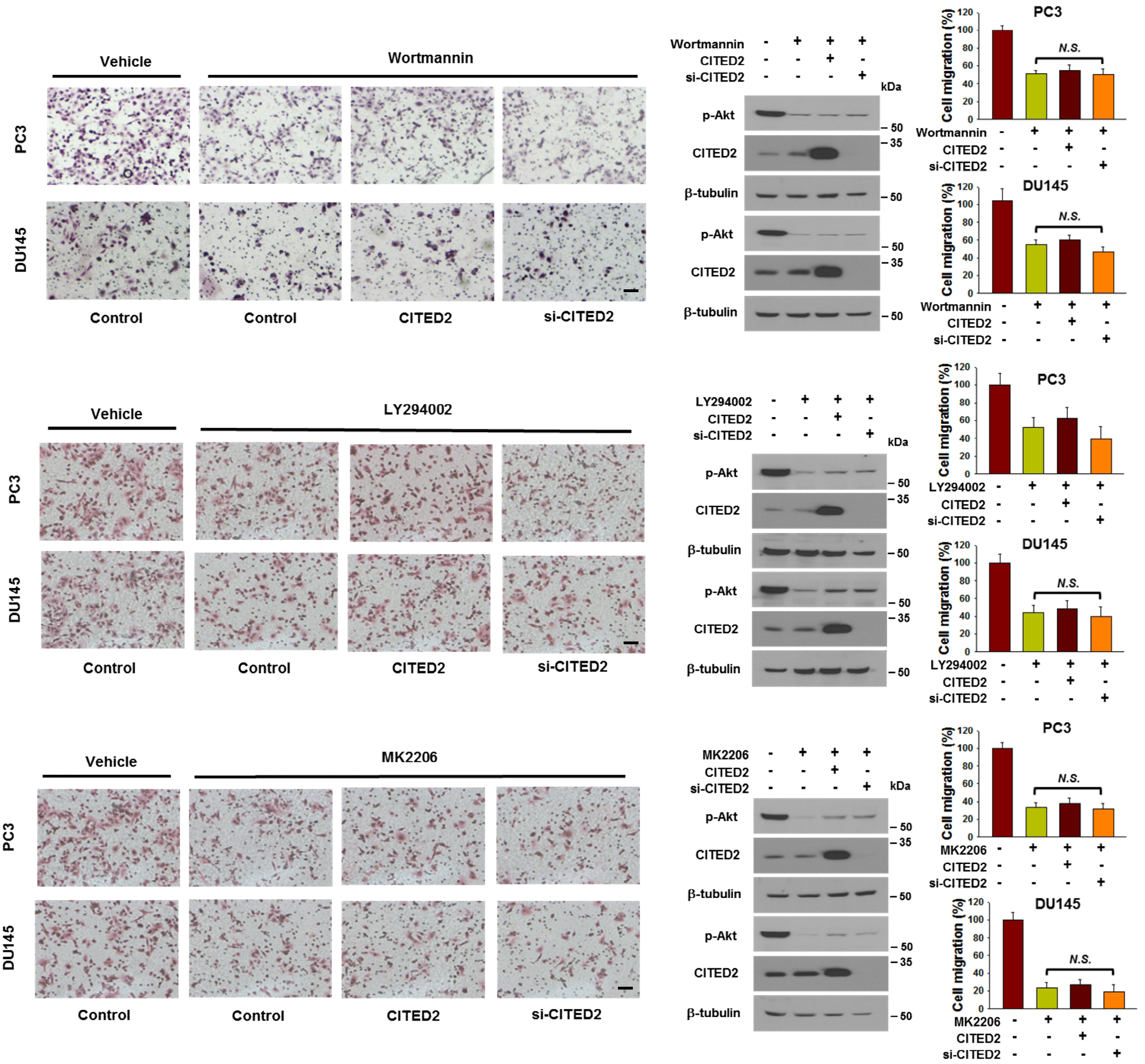

Supplementary Fig. 11 CITED2-activated NCL promotes the AKT-driven cell migration. Representative pictures of trans-well migration assays. The transfected cells were subjected to the cell migration assay in the presence of wortmannin (200 nM), LY294002 (25 μM), or MK2206 (2 μM). CITED2 and phospho-AKT levels were analyzed by Western blotting. Migrated cells were stained and counted, which are presented as bar graphs (means + s.d., n=3). N.S. represents 'not significantly different' among the indicated groups by Student's t-test. The scale bar represents 25 μm.

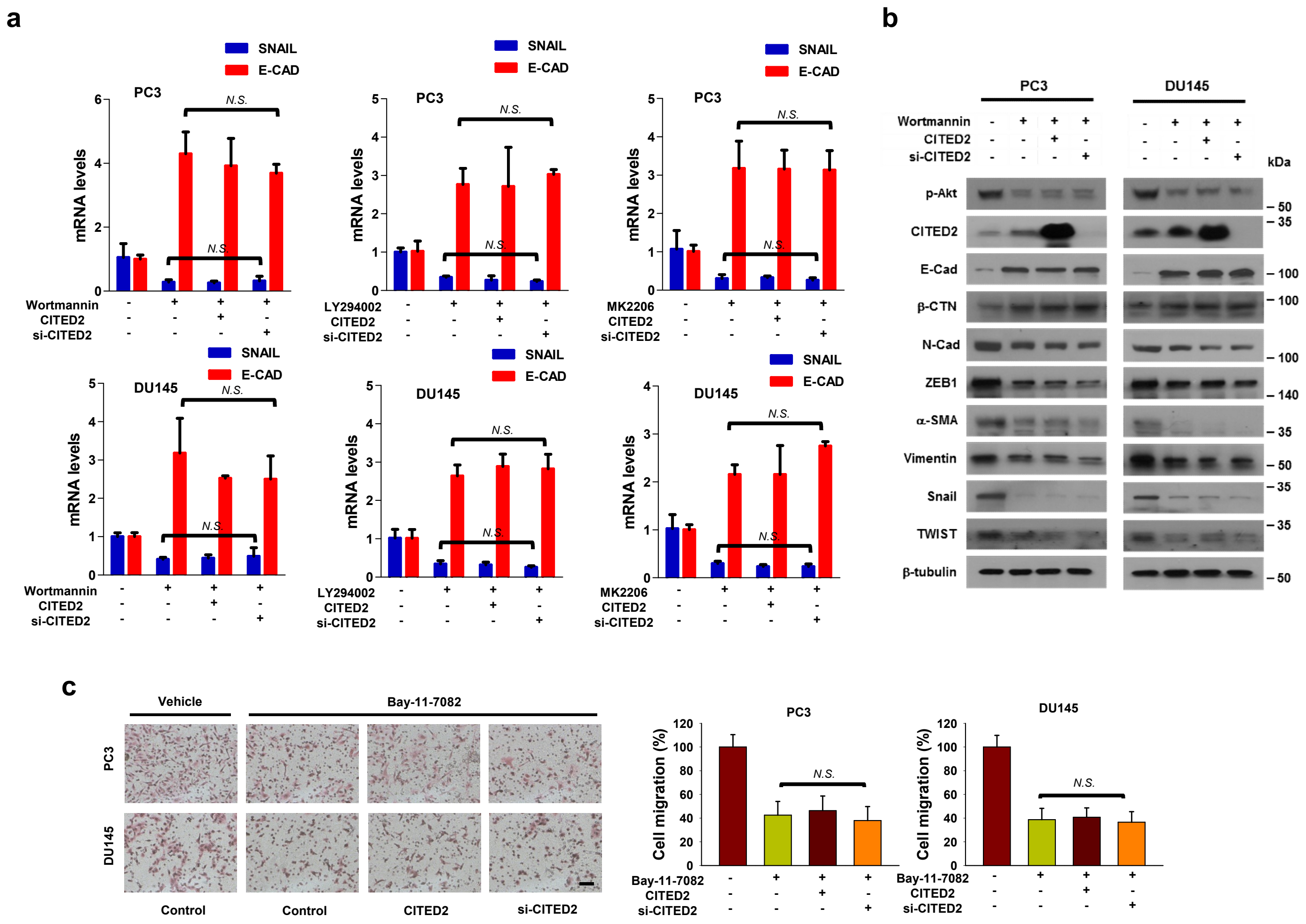

Supplementary Fig. 12 CITED2 regulates the EMT-related gene expression via the NF-kB pathway. **a** PC3 and DU145 cells, which had been transfected with CITED2 or si-CITED2, were treated with wortmannin (200 nM), LY294002 (25 μM), or MK2206 (2 μM). The *SNAIL* and *E-CAD* mRNA levels (means + s.d., n=3) were measured by RT-qPCR. N.S. represents 'not significantly different' among the indicated groups by Student's t-test. **b** EMT markers were immunoblotted in PC3 and DU145 cells that were transfected with CITED2 or si-CITED2 and treated with wortmannin (200 nM). **c** Representative pictures of trans-well migration assays. The transfected cells were subjected to the cell migration assay in the presence of Bay 11-7082 (10 μM). Migrated cells were stained and counted, which are presented as bar graphs (means + s.d., n=3). N.S. represents 'not significantly different' among the indicated groups by Student's t-test. The scale bar represents 25 μm.

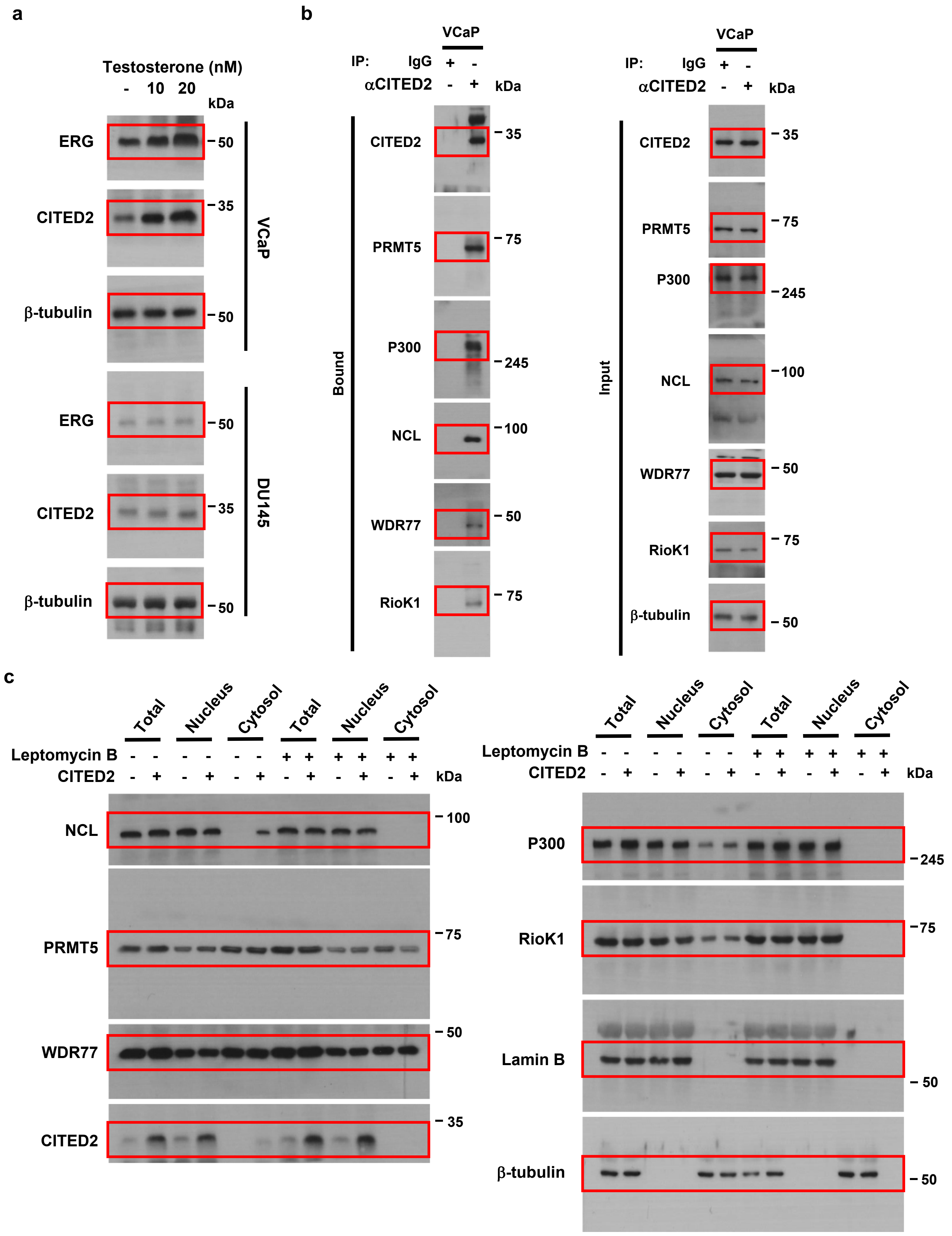

Supplementary Fig. 13 Original images of Western blots used in the study. **a** Uncropped blots show indicated proteins and molecular weight markers from Figure 2d. **b** Uncropped blots show indicated proteins and molecular weight markers from Figure 3b. **c** Uncropped blots show indicated proteins and molecular weight markers from Figure 4a. The red boxes indicate cropped blots.

Supplementary Table 1. List of antibodies used in immunoblotting.

Antibodies	SOURCE	Catalog #	Dilution
CITED2	Santa Cruz Biotechnology	Cat.sc-21795	1 : 1000
PRMT5	Santa Cruz Biotechnology	Cat.sc-22132	1 :1000
β-tubulin	Santa Cruz Biotechnology	Cat.sc-9104	1: 2000
WDR77	Santa Cruz Biotechnology	Cat.sc-100899	1 : 1000
RioK1	Santa Cruz Biotechnology	Cat.sc-130253	1 : 500
P300	Santa Cruz Biotechnology	Cat.sc-32244	1 : 1000
β-CTN	Santa Cruz Biotechnology	Cat.sc-7963	1 : 2000
Vimentin	Santa Cruz Biotechnology	Cat.sc-7558	1 : 1000
TWIST	Santa Cruz Biotechnology	Cat.sc-15393	1 : 1000
Snail	Santa Cruz Biotechnology	Cat.sc-28199	1 : 1000
N-Cad	Santa Cruz Biotechnology	Cat.sc-7939	1 : 1000
ZEB1	Santa Cruz Biotechnology	Cat.sc-25388	1 : 1000
Acetyl-lysine	Cell Signaling Technology	Cat.#9441	1 : 500
p-Akt	Cell Signaling Technology	Cat.#4060	1 : 2000
Akt	Cell Signaling Technology	Cat.#9272	1 : 2000
E-Cad	Cell Signaling Technology	Cat.#3195	1 : 2000
Myc	Cell Signaling Technology	Cat.#2276	1 : 2000
ERG	Abcam	Cat.Ab110639	1 : 1000
α-SMA	Abcam	Cat.Ab5694	1 : 1000
NCL	Upstate	Cat.05-565	1 : 1000
Dimethyl-arginine	EMD millipore	Cat.07-412	1 : 500
FLAG	Sigma	Cat.F3165	1 : 2000
HA	Roche Life Science	Cat.11867423001	1 : 1000

Supplementary Table 2. Clinical information on prostate cancer patients.

No.	Age	Sex	Organ	Diagnosis	Gleason score	Stage	Residual tumor	Follow-up month	Live	PSA (ng/ml)
1	60	Male	Prostate	adenocarcinoma	9	3	no	60	alive	11.2
2	64	Male	Prostate	adenocarcinoma	7	2b	no	60	alive	30
3	71	Male	Prostate	adenocarcinoma	9	3	yes	55	alive	60.4
4	64	Male	Prostate	adenocarcinoma	10	3	yes	47	alive	7.4
5	59	Male	Prostate	adenocarcinoma	9	3	yes	44	alive	9.8
6	65	Male	Prostate	adenocarcinoma	8	3	yes	43	alive	34.9
7	73	Male	Prostate	adenocarcinoma	7	2b	yes	42	alive	48.1
8	69	Male	Prostate	adenocarcinoma	7	2b	no	42	alive	10.6
9	62	Male	Prostate	adenocarcinoma	7	2b	yes	39	alive	37.3
10	66	Male	Prostate	adenocarcinoma	9	3	yes	39	alive	1.2
11	60	Male	Prostate	adenocarcinoma	9	3	yes	39	alive	40
12	66	Male	Prostate	adenocarcinoma	7	3	yes	39	alive	8.4
13	70	Male	Prostate	adenocarcinoma	7	3	yes	37	alive	7
14	65	Male	Prostate	adenocarcinoma	9	3	yes	23	dead	17.5
15	67	Male	Prostate	adenocarcinoma	9	3	yes	34	alive	13.1
16	69	Male	Prostate	adenocarcinoma	7	3	yes	33	alive	1.1
17	63	Male	Prostate	adenocarcinoma	9	3	yes	33	alive	11.8
18	69	Male	Prostate	adenocarcinoma	7	3	yes	27	alive	17.6
19	70	Male	Prostate	adenocarcinoma	7	3	yes	26	alive	9
20	58	Male	Prostate	adenocarcinoma	9	3	no	26	alive	5.8
21	58	Male	Prostate	adenocarcinoma	7	3	yes	24	alive	15.8
22	71	Male	Prostate	adenocarcinoma	7	2b	yes	24	alive	31.4
23	70	Male	Prostate	adenocarcinoma	7	3	yes	19	alive	14.4
24	59	Male	Prostate	adenocarcinoma	6	2a	no	18	alive	18.3
25	63	Male	Prostate	adenocarcinoma	9	3	yes	17	alive	16.6
26	72	Male	Prostate	adenocarcinoma	9	3	yes	16	alive	.
27	66	Male	Prostate	adenocarcinoma	8	2	yes	17	dead	10.8
28	70	Male	Prostate	adenocarcinoma	6	3	yes	16	alive	10.8
29	70	Male	Prostate	adenocarcinoma	7	2b	no	15	alive	.
30	68	Male	Prostate	adenocarcinoma	8	3	yes	15	alive	26.9
31	63	Male	Prostate	adenocarcinoma	10	3	yes	15	alive	.
32	57	Male	Prostate	adenocarcinoma	7	3	yes	15	alive	25
33	72	Male	Prostate	adenocarcinoma	8	2b	yes	15	alive	16.8
34	70	Male	Prostate	adenocarcinoma	8	3	yes	15	alive	0.5
35	75	Male	Prostate	adenocarcinoma	9	3	yes	15	alive	98
36	62	Male	Prostate	adenocarcinoma	9	3	yes	15	alive	.
37	63	Male	Prostate	adenocarcinoma	9	3	yes	14	alive	91
38	53	Male	Prostate	adenocarcinoma	9	3	yes	17	dead	161
39	63	Male	Prostate	adenocarcinoma	8	3	yes	13	alive	13
40	44	Male	Prostate	adenocarcinoma	7	3	yes	11	alive	.
41	69	Male	Prostate	normal	.	.	.	.	.	.
42	62	Male	Prostate	normal	.	.	.	.	.	.
43	66	Male	Prostate	normal	.	.	.	.	.	.
44	65	Male	Prostate	normal	.	.	.	.	.	.
45	69	Male	Prostate	normal	.	.	.	.	.	.
46	70	Male	Prostate	normal	.	.	.	.	.	.
47	70	Male	Prostate	normal	.	.	.	.	.	.
48	63	Male	Prostate	normal	.	.	.	.	.	.
49	44	Male	Prostate	normal	.	.	.	.	.	.

Supplementary Table 3. Sequences of primers used in PCR and ChIP.

Gene	Direction	Nucleotide Sequence
E-CADHERIN	Forward	5′-ACAACAAGCCCGAATTCACCCA-3′
	Reverse	5′-TCACAGCTGTTGCTGTTGTGCT-3′
SNAIL	Forward	5′-CCCAATCGGAAGCCTAACTA-3′
	Reverse	5′-CAGGACAGAGTCCCAGATGAG-3′
GAPDH	Forward	5′-TGTGGTCATGAGTCCTTCCA-3’
	Reverse	5’-CGAGATCCCTCCAAAATCAA-3’
CITED2	Forward	5’-ACCCACCTCCCTTATGTAGT-3’
	Reverse	5’-CCAACTAATGCAATTTTTCC-3’
AKT	Forward	5’-GCCATGAAGATCCTCAAGAA-3’
	Reverse	5’-GTACTCCATGACAAAGCAGA-3’
TMPRSS2-ERG	Forward	5’-CAGGAGGCGGAGGCGGA-3’
	Reverse	5’-GGCGTTGTAGCTGGGGGTGAG-3’
CITED2-P1	Forward	5’-CACTGCCCTGATCTTTCTAA-3’
	Reverse	5’-TGGGGTAGAAAATGTATGGC-3’
CITED2-P2	Forward	5’-GACTAGGAAAAGACAGAGGG-3’
	Reverse	5’-ACTCGATAGCCATTGAAGAC-3’
CITED2-P3	Forward	5’-ATGAAAACACAAAGGCACAG-3’
	Reverse	5’-TTTCTCACTCGGTTCACCTA-3’
