# Supplementary material for: Aberrant expression of CITED2 promotes prostate cancer metastasis by activating the nucleolin-AKT pathway
Source: Nat Commun. 2018 Oct 5;9:4113. doi: 10.1038/s41467-018-06606-2 (PMC6173745; doi:10.1038/s41467-018-06606-2)
Supplement: Supplementary file 4 — Description of Additional Supplementary Files [file 41467_2018_6606_MOESM4_ESM.docx]

**Title:** Supplementary Data 1

**Description:** List of CITED2-interacting proteins using LC-MS/MS. HEK293T cells were transfected with Flag/SBP-CITED2, and proteins in cell lysates were precipitated by anti-Flag (redcolored) or streptavidin (blue-colored) affinity beads. Co-precipitated proteins were identified using LC-MS/MS. The proteins purified commonly by both affinity beads are listed.
